# Supplementary material for: MetaRibo-Seq measures translation in microbiomes
Source: Nat Commun. 2020 Jun 29;11:3268. doi: 10.1038/s41467-020-17081-z (PMC7324362; doi:10.1038/s41467-020-17081-z)
Supplement: Supplementary file 10 — Supplementary Data 7 [file 41467_2020_17081_MOESM10_ESM.zip › File2/Confidence_High_Taxonomy/395444_out.krona.html]

Javascript must be enabled to view this page.

members
magnitude
magnitudeUnassigned
count
unassigned
taxon
rank

395444\_out

215

superkingdom
2
215

215
phylum
976

class
200643
215

171549
order
215

family
171550
1

239759
genus
1

214856
species

SRS893342\_contig\_number\_13
1

815
family
212

genus

SRS013951\_contig\_number\_contig-100\_1282.215107SRS014459\_contig\_number\_7340SRS014683\_contig\_number\_714SRS015065\_contig\_number\_18403SRS015190\_contig\_number\_12229SRS015264\_contig\_number\_6232SRS015431\_contig\_number\_67796SRS015794\_contig\_number\_3910SRS015854\_contig\_number\_21356SRS018984\_contig\_number\_73SRS020869\_contig\_number\_10781SRS021484\_contig\_number\_20202SRS024435\_contig\_number\_32534SRS043411\_contig\_number\_15576SRS045739\_contig\_number\_7890SRS050299\_contig\_number\_1156SRS050752\_contig\_number\_2592SRS053398\_contig\_number\_12298SRS054352\_contig\_number\_11533SRS055982\_contig\_number\_6369SRS063813\_contig\_number\_6391SRS065176\_contig\_number\_4322SRS074964\_contig\_number\_1251SRS075821\_contig\_number\_21735SRS076976\_contig\_number\_18475SRS077024\_contig\_number\_contig-100\_181.104358SRS077502\_contig\_number\_5449SRS078242\_contig\_number\_19012SRS078665\_contig\_number\_592SRS097889\_contig\_number\_950SRS098644\_contig\_number\_11537SRS098644\_contig\_number\_11710SRS104084\_contig\_number\_contig-100\_593.73855SRS1041038\_contig\_number\_14371SRS1041095\_contig\_number\_5646SRS104975\_contig\_number\_14721SRS140645\_contig\_number\_590SRS143372\_contig\_number\_6489SRS143895\_contig\_number\_4751SRS144506\_contig\_number\_56534SRS144537\_contig\_number\_23055SRS147614\_contig\_number\_1995SRS147653\_contig\_number\_7792SRS148196\_contig\_number\_39917SRS149181\_contig\_number\_16008SRS893231\_contig\_number\_3302SRS893327\_contig\_number\_9732SRS893341\_contig\_number\_639SRS893378\_contig\_number\_19671SRS893382\_contig\_number\_1191SRS893383\_contig\_number\_23997
816
51
212

2

SRS075773\_contig\_number\_15929SRS148511\_contig\_number\_2358
species
246787

species
2292944
1

SRS147346\_contig\_number\_16819

species
162156
1

SRS045244\_contig\_number\_6969

338188
species
2

SRS013521\_contig\_number\_814SRS140513\_contig\_number\_5610

28116
species

SRS012902\_contig\_number\_87SRS013215\_contig\_number\_1488SRS013382\_contig\_number\_2389SRS014855\_contig\_number\_10721SRS014979\_contig\_number\_contig-100\_92.201868SRS015217\_contig\_number\_4304SRS015578\_contig\_number\_28205SRS015663\_contig\_number\_13676SRS016132\_contig\_number\_1684SRS016203\_contig\_number\_5215SRS016381\_contig\_number\_2755SRS016753\_contig\_number\_3260SRS016840\_contig\_number\_1598SRS016989\_contig\_number\_1901SRS017103\_contig\_number\_13775SRS018623\_contig\_number\_3825SRS019068\_contig\_number\_34444SRS019381\_contig\_number\_1675SRS019693\_contig\_number\_7107SRS022137\_contig\_number\_17134SRS022713\_contig\_number\_997SRS023715\_contig\_number\_20045SRS023829\_contig\_number\_63SRS043001\_contig\_number\_7357SRS044535\_contig\_number\_11048SRS049959\_contig\_number\_33595SRS050941\_contig\_number\_8800SRS058268\_contig\_number\_136SRS064232\_contig\_number\_contig-100\_423.38615SRS074670\_contig\_number\_4585SRS075398\_contig\_number\_118SRS077086\_contig\_number\_3452SRS077194\_contig\_number\_11834SRS077849\_contig\_number\_9873SRS097958\_contig\_number\_18398SRS098571\_contig\_number\_15840SRS1041092\_contig\_number\_9897SRS1041134\_contig\_number\_2441SRS1041147\_contig\_number\_3106SRS104485\_contig\_number\_218SRS1055049\_contig\_number\_10023SRS1055056\_contig\_number\_9996SRS1055095\_contig\_number\_732SRS142503\_contig\_number\_31671SRS143085\_contig\_number\_878SRS143466\_contig\_number\_18913SRS144183\_contig\_number\_contig-100\_192.219153SRS147088\_contig\_number\_2144SRS148159\_contig\_number\_21015SRS148319\_contig\_number\_2886SRS148970\_contig\_number\_1417SRS149075\_contig\_number\_11992SRS149244\_contig\_number\_106SRS150029\_contig\_number\_10977SRS893253\_contig\_number\_229SRS893358\_contig\_number\_10552
56

6

SRS016095\_contig\_number\_30391SRS045244\_contig\_number\_1001SRS056537\_contig\_number\_47164SRS062701\_contig\_number\_2294SRS1041142\_contig\_number\_721SRS1055038\_contig\_number\_76
species
457390

1

SRS024388\_contig\_number\_8939
species
671267

2292956
species

SRS013476\_contig\_number\_12867SRS013940\_contig\_number\_6455SRS014613\_contig\_number\_1807SRS017622\_contig\_number\_75SRS017859\_contig\_number\_6157SRS018133\_contig\_number\_3203SRS018427\_contig\_number\_14106SRS018936\_contig\_number\_1643SRS020394\_contig\_number\_12820SRS043701\_contig\_number\_11188SRS057480\_contig\_number\_9225SRS063489\_contig\_number\_6222SRS064276\_contig\_number\_8112SRS076804\_contig\_number\_18853SRS1041037\_contig\_number\_10306SRS1041164\_contig\_number\_25SRS1055067\_contig\_number\_13540SRS1055069\_contig\_number\_4583SRS147977\_contig\_number\_4257SRS148721\_contig\_number\_691
20

species
820

SRS019968\_contig\_number\_15981SRS098061\_contig\_number\_7833
2


SRS1041136\_contig\_number\_16518
1
1946086
species

species
376806
1

SRS046068\_contig\_number\_1138

2292280
species
1

SRS098881\_contig\_number\_6244

821
species
3

SRS015579\_contig\_number\_3537SRS023346\_contig\_number\_11SRS063190\_contig\_number\_1648


SRS1055076\_contig\_number\_11741
1
species
1263050


SRS014287\_contig\_number\_10880SRS019397\_contig\_number\_1173SRS062427\_contig\_number\_403
3
species
2292957

371601
species
8

SRS013687\_contig\_number\_12801SRS020271\_contig\_number\_2475SRS023914\_contig\_number\_13596SRS053573\_contig\_number\_453SRS098655\_contig\_number\_8931SRS142980\_contig\_number\_5370SRS144297\_contig\_number\_contig-100\_327.77478SRS148784\_contig\_number\_6123

817
species
1

SRS057717\_contig\_number\_367

1262750
species

SRS063928\_contig\_number\_278SRS893256\_contig\_number\_719
2

species
2292938
1

SRS097920\_contig\_number\_14246

1739517
species

SRS014736\_contig\_number\_5171SRS018351\_contig\_number\_17647SRS020508\_contig\_number\_236SRS022524\_contig\_number\_15253SRS045645\_contig\_number\_991SRS045826\_contig\_number\_11628SRS046717\_contig\_number\_1888SRS046810\_contig\_number\_contig-100\_148.149SRS048870\_contig\_number\_4597SRS052576\_contig\_number\_877SRS054905\_contig\_number\_26344SRS054956\_contig\_number\_5335SRS056519\_contig\_number\_1172SRS065397\_contig\_number\_11372SRS098717\_contig\_number\_6883SRS101376\_contig\_number\_7104SRS1041141\_contig\_number\_4365SRS142923\_contig\_number\_19505SRS143523\_contig\_number\_8202SRS143991\_contig\_number\_29395SRS145308\_contig\_number\_4324SRS146813\_contig\_number\_12162SRS147919\_contig\_number\_1648SRS148424\_contig\_number\_4248SRS892882\_contig\_number\_16470SRS893259\_contig\_number\_6394SRS893373\_contig\_number\_25290
27

species
46506
3

SRS049995\_contig\_number\_11579SRS057478\_contig\_number\_110SRS893288\_contig\_number\_422

1

SRS020328\_contig\_number\_14152
species
2292939

585544
species
1

SRS020622\_contig\_number\_422


SRS013638\_contig\_number\_3551SRS077552\_contig\_number\_12197SRS078419\_contig\_number\_2403SRS1054928\_contig\_number\_850
4
556259
species

4

SRS018575\_contig\_number\_7206SRS023583\_contig\_number\_1287SRS042966\_contig\_number\_contig-100\_1054.124842SRS148091\_contig\_number\_508
818
species

329854
species

SRS015960\_contig\_number\_10278
1

species
47678
7

SRS018313\_contig\_number\_308SRS019445\_contig\_number\_35071SRS051882\_contig\_number\_8376SRS058770\_contig\_number\_14193SRS1041157\_contig\_number\_6668SRS104311\_contig\_number\_8086SRS1055083\_contig\_number\_8455

family
171552
2

577309
genus
2

1

SRS017433\_contig\_number\_contig-100\_492.106259
454154
species

species
1263095
1

SRS1055022\_contig\_number\_13045
